# Inhibition deficit in the spatial tendency of the response in multiple-domain amnestic mild cognitive impairment. An event-related potential study

**DOI:** 10.3389/fnagi.2015.00068

**Published:** 2015-05-06

**Authors:** Jesús Cespón, Santiago Galdo-Álvarez, Fernando Díaz

**Affiliations:** Cognitive Neuroscience Laboratory, Facultade de Psicoloxía, Universidade de Santiago de CompostelaSantiago de Compostela, Spain

**Keywords:** event-related potentials (ERP), mild cognitive impairment (MCI), negativity central contralateral (N2cc), negativity posterior contralateral (N2pc), inhibitory control, stimulus-response compatibility tasks (SRC)

## Abstract

Longitudinal studies have shown that a high percentage of people with amnestic mild cognitive impairment (MCI) develop Alzheimer’s disease (AD). Prodromal AD is known to involve deficits in executive control processes. In the present study, we examined such deficits by recording EEG in 13 single-domain amnestic MCI (sdaMCI), 12 multiple-domain amnestic MCI (mdaMCI) and 18 healthy elderly (control group, CG) participants while they performed a Simon task. The Simon task demands deployment of executive processes because participants have to respond to non-spatial features of a lateralized stimulus and inhibit the more automatic spatial tendency of the response. We specifically focused on the negativity central contralateral (N2cc), an event-related potential (ERP) component related to brain activity that prevents the cross-talk between direction of spatial attention and manual response preparation. The reaction time (RT) was not significantly different among the three groups of participants. The percentage of errors (PE) was higher in mdaMCI than in CG and sdaMCI participants. In addition, N2cc latency was delayed in mdaMCI (i.e., delayed implementation of mechanisms for controlling the spatial tendency of the response). The N2cc latency clearly distinguished among mdaMCI and CG/sdaMCI participants (area under curve: 0.91). Longer N2cc was therefore associated with executive control deficits, which suggests that N2cc latency is a correlate of mdaMCI.

## Introduction

Mild Cognitive Impairment (MCI) is a diagnostic entity applied to patients with symptoms suggestive of Alzheimer’s disease (AD) but which are not sufficient to interfere with the daily routine (Petersen et al., 1999). Studies have demonstrated that a high percentage of such patients develop dementia within a few years, and MCI is therefore considered a prodromal stage of AD (Petersen et al., 2009). Nonetheless, the clinical symptoms and prognosis are heterogeneous (Petersen et al., 2009). Thus, MCI is divided into four subtypes according to the presence/absence of episodic memory impairment and deficits on single or multiple cognitive domains (Petersen, 2004; Winblad et al., 2004). Subsequent studies have associated both amnestic subtypes and particularly the multiple-domain amnestic MCI subtype (mdaMCI) with poorer prognosis and higher rates of conversion to AD (Fischer et al., 2007; Hunderfund et al., 2006; Petersen and Negash, 2008).

Considering the evidence above, correlates of brain activity in single-domain amnestic MCI (sdaMCI) and especially in mdaMCI may represent early AD biomarkers. Identification of such biomarkers would allow early intervention to slow progression of the disease (Levey et al., 2006). Electroencephalography (EEG) and event-related brain potential (ERP) techniques are considered suitable methods for obtaining biomarkers because they are non-invasive, inexpensive and widely used procedures (Rossini et al., 2006). The temporal resolution of these techniques is also high, which is essential for dealing with the high speed of the cognitive processes and underlying mechanisms that usually are affected in prodromal AD (Barrett, 2000).

Deficits in cognitive control functions are observed very early in AD (for reviews, see Perry and Hodges, 1999; Amieva et al., 2004). In fact, some studies have revealed that MCI participants already show deficits in cognitive control (Rosano et al., 2005; Johns et al., 2012). Such deficits have been indicated by increased interference from irrelevant information in the performance of several cognitive tasks, such as stimulus-response compatibility (SRC) tasks. SRC tasks demand inhibition of a more automatic response in favor of an alternative response based on task instructions (Zhang et al., 1999; Wylie et al., 2007; Pereiro et al., 2014).

The Simon task has been proposed as a suitable SRC task for studying cognitive control deficits (Simon, 1990; Leuthold, 2011). Specifically, in the Simon task, participants are required to respond to spatially lateralized stimuli by pressing one of two buttons. The response buttons are also lateralized in the same spatial arrangement as the stimuli, with the position of the stimuli being irrelevant to the task. In those cases in which the required response is on the opposite side to the stimulus (incompatible condition), a type of interference known as the Simon effect is observed; this leads to increased reaction time (RT) and percentage of errors (PE) (for reviews, see Proctor et al., 2005; Leuthold, 2011). Several studies have shown that interference in Simon tasks increases with age of participants (van der Lubbe and Verleger, 2002; Proctor et al., 2005; Juncos-Rabadán et al., 2008) and it is even greater in participants with MCI (Cespón et al., 2013a; Pereiro et al., 2014). However, changes in neural mechanisms underlying cognitive control processes in Simon tasks have not been studied in MCI persons.

The negativity central contralateral (N2cc) is an ERP component associated with cognitive control in Simon-type tasks. The N2cc is obtained by a subtraction procedure that isolates increased activity occurring between 200–250 ms after stimulus presentation at central electrode sites contralateral to the hemifield in which the target stimulus is presented (Oostenveld et al., 2001; Praamstra and Plat, 2001). Evidence from intracranial recordings in studies involving non-human primates (Wise et al., 1996, 1997; Crammond and Kalaska, 2000), neuroimaging (Connolly et al., 2000; Dassonville et al., 2001) and ERP recordings (Oostenveld et al., 2001; Praamstra and Oostenveld, 2003; Praamstra, 2006; Cespón et al., 2012) have associated N2cc with activity from the dorsal premotor cortex (dPM) involved in preventing the cross-talk between the direction of the spatial attention and preparation of the manual response.

In a recent study, we observed altered spatial attention toward a lateralized target stimulus, as revealed by the smaller amplitude of the negativity posterior contralateral (N2pc) in mdaMCI participants than in healthy controls (Cespón et al., 2013a). N2pc is an ERP correlate of the direction of the spatial attention to lateralized stimuli (Luck and Hillyard, 1994; Woodman and Luck, 1999; Hickey et al., 2009). N2pc is obtained using the same procedure than N2cc but at posterior electrode sites (Eimer, 1996). The neural sources of N2pc have been localized in extrastriate visual areas (Luck et al., 1997; Hopf et al., 2000). Considering the above-mentioned deficits in allocation of attentional resources to target stimulus together with the increased interference exhibited by mdaMCI participants in Simon-type tasks (Rosano et al., 2005; Johns et al., 2012; Cespón et al., 2013a; Pereiro et al., 2014), we can hypothesize that mechanisms related to prevent the cross-talk between the direction of the spatial attention and the response preparation (i.e., the N2cc correlate) may be affected in mdaMCI. Also, some studies suggest that deficits in brain mechanisms precede behavioral manifestations (Galli et al., 2010; Vallesi and Stuss, 2010). Therefore, N2cc parameters may already be affected in sdaMCI participants even though performance is similar in the sdaMCI and the control groups.

A previous study concerning age-related changes in N2cc activity found longer N2cc latencies and increased ratio of N2cc/N2pc amplitudes in healthy elderly adults than in young adults (Amenedo et al., 2012). The higher ratio of N2cc/N2pc amplitudes was associated with increased direct visuomotor transmission from parietal to central areas (van der Lubbe and Verleger, 2002; Amenedo et al., 2012). That increased ratio was also related to disinhibitory processes that led to increased cross-talk between the direction of spatial attention and manual response preparation (van der Lubbe and Verleger, 2002; Amenedo et al., 2012). As already mentioned, the N2cc component has not been studied in relation to MCI. Therefore, the main aim of the present study was to use a Simon task to examine possible differences in N2cc (the ERP correlate of the ability to prevent spatial tendencies of response) between participants diagnosed with amnestic MCI (distinguishing mdaMCI and sdaMCI subtypes) and healthy control participants.

Considering previous results (Cespón et al., 2013a), behavioral performance was expected to differ between healthy control and mdaMCI but not between healthy control and sdaMCI participants. Specifically, we expected increased interference in mdaMCI relative to healthy control and sdaMCI participants, as reflected by a higher PE and/or longer RTs when the target stimulus is spatially incompatible with the required response than in the condition where the target stimulus is spatially compatible with the required response.

In accordance with the behavioral hypothesis above, increased direct visuomotor transmission from parietal to central areas might be expected in mdaMCI relative to healthy participants, as reflected by higher ratio of N2cc/N2pc amplitudes in mdaMCI than in healthy elderly participants. On the other hand, no differences in N2cc parameters between CG and sdaMCI participants would be in line with expected behavioral performance (that is, no differences in interference effect between both groups). Alternatively, differences between sdaMCI and healthy participants in N2cc parameters may reflect incipient executive control decline in sdaMCI participants, regardless of whether this is apparent in the behavioral performance.

## Method

### Participants

Forty-three participants (26 women, 17 men) between 60 and 83 years of age were recruited from the general population. Participants were divided into three groups: 18 healthy participants (eleven women; mean age: 68.3), 13 participants diagnosed with single-domain amnestic MCI (sdaMCI) (five women; mean age: 69.1) and 12 participants diagnosed with multiple-domain amnestic MCI (mdaMCI) (seven women; mean age: 71.2). All participants were right-handed (evaluated by the Edinburgh Handedness Inventory: Oldfield (1971)) and had normal or corrected to normal vision. Participants did not have any history of neurological or psychiatric disorders. Experimental procedures and aims of the research were explained and all the participants gave written informed consent prior to their inclusion in the study. When a relative accompanied the participant, both were present when the tasks and the aims of the research were explained. The study received prior approval by the USC ethics committee and by the Galician Clinical Research ethics committee. All potential participants who declined to participate were not disadvantaged in any way by not participating in the study.

The following tests were used to diagnose sdaMCI and mdaMCI: an adapted version (Lobo et al., 1999) of the Mini-mental state Examination (MMSE; Folstein et al., 1975); an adapted version (TAVEC, Test de Aprendizaje Verbal España Complutense; Benedet Álvarez and Alexandre, 1998) of the California Verbal Learning Test (Delis et al., 1987); the Cambridge examination for mental disorders in elderly (CAMDEX-r) (Roth et al., 1986); a questionnaire on subjective memory complaints (Benedet and Seisdedos, 1996); the instrumental activities of daily living scale (IADL; Lawton and Brody, 1969); and the Geriatric depression scale (GDS; Sheikh and Yesavage, 1986). Participants also completed a questionnaire with socio-demographic and clinical data. The groups did not differ according to years of schooling or age. Moreover, correlations between years of schooling and the ERP parameters and between WAIS scores and the ERP parameters were not significant. These analyses excluded possible influence of cultural level on correlates of the studied cognitive processes, which is consistent with consideration of Simon-type tasks as not being influenced by cultural level (Simon, 1990).

The sdaMCI and mdaMCI participants fulfilled the following criteria: (1) memory complaints corroborated by an informant; (2) performance of less than 1.5 standard deviations (SDs) below age norms for the TAVEC; (3) no significant impact on activities of daily living; and (4) not suffering from dementia; mdaMCI participants also fulfilled a fifth criterion as regards general cognitive functioning, i.e., they scored less than 1.5 SDs below controls with respect to standards of age and years of schooling, in the adapted version of the MMSE, and on at least two cognitive subscales of the Spanish version of the Cambridge Cognitive Examination -CAMCOG-R- (a subscale of the CAMDEX-r). All control participants scored higher than cut-off on memory, general cognitive functioning and specific cognitive domain tests (scores in the more relevant demographic and neuropsychological measures are summarized in Table 1). For an extensive description of the samples, the inclusion/exclusion criteria, the tests used and the diagnostic/classification criteria, see Juncos-Rabadán et al. (2013).

**Table 1:** **Mean values ± standard error of the mean (SEM, in parentheses) of the main demographic and neuropsychological measures**.

	CG	sdaMCI	mdaMCI
Age	68.3 (1.68)	69.1 (1.98)	71.2 (2.06)
Schooling	11.1 (1.24)	10.1 (1.46)	9.2 (1.52)
WAIS_language	52.6 (3.38)	47.9 (3.98)	40.7 (4.15)
CAMCOG_MMSE	28.5 (0.38)	26.9 (0.45)	23.7 (0.47)*
CAMCOG_orientation	9.6 (0.17)	9.3 (0.21)	8.5 (0.21)**
CAMCOG_language	26.4 (0.55)	25.2 (0.65)	23.2 (0.67)*
CAMCOG_calculation	7.6 (0.43)	6.8 (0.51)	5.0 (0.53)**
CAMCOG_praxis	11.4 (0.34)	10.7 (0.40)	9.4 (0.42)**
CAMCOG_perception	6.5 (0.38)	6.3 (0.45)	6.3 (0.47)
CAMCOG_executive	19.6 (1.00)	16.5 (1.18)	13.5 (1.23)**
CVLT (short delay free recall)	10.0 (0.52)	6.2 (0.62)*	3.5 (0.64)*
CVLT (short delay cue recall)	11.0 (0.56)	7.6 (0.66)*	5.7 (0.69)*
CVLT (long delay free recall)	10.5 (0.66)	6.7 (0.78)*	3.2 (0.81)*
CVLT (long delay cue recall)	11.4 (0.65)	8.2 (0.76)*	5.4 (0.79)*

*Univariate ANOVAs (Diagnosis) were carried out to compare scores at group level among groups of participants (healthy control group (CG), single-domain amnestic MCI (sdaMCI), and multiple-domain amnestic MCI (mdaMCI)) on each cognitive scale. One asterisk indicates significant differences (*p* < 0.05) between that particular MCI group regarding the CG. Two asterisks indicate significant differences between mdaMCI group regarding CG and sdaMCI group*.

### Task and Procedure

The task used in the present study was the same that we used in a previous study (for further details about the stimuli used, see Cespón et al., 2013a, Figure 1). In each trial, a red or blue arrow pointing either left or right was displayed on a screen against a black background. The screen was placed in front of the participants. The distance between the participants and the screen was 100 cm. The arrow stimuli subtended 2.87° long and 1.72° wide of the visual field. The visual angles between the small central fixation cross in the middle of the screen and the internal and external edges of the arrow were 2.29° and 5.16° respectively, so that the whole stimulus was presented in the parafoveal region (Bargh and Chartrand, 2000). In order to prevent exogenous lateralization in the EEG, a gray figure of similar size and eccentric position was presented in the contralateral hemifield regarding the arrow position. The stimulation (i.e., arrow and contralateral non target stimulus) was presented for 125 ms, with 2000 ms inter-trial intervals. The participants were instructed to direct their gaze towards the central cross, which remained in the center of the screen throughout the task. This, together with the short time during which stimuli were presented, minimized the likelihood of ocular movements towards the arrow position (Abrahamse and Van der Lubbe, 2008).

**Figure 1:**
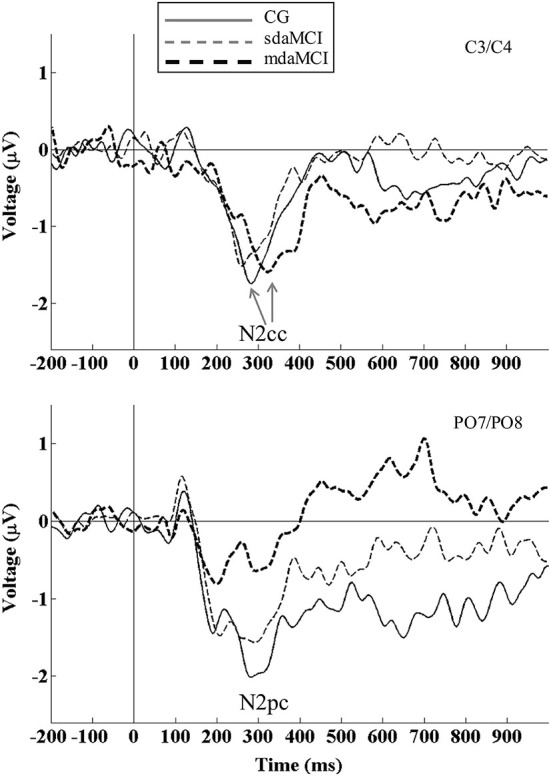
**Event-related potential waveforms: N2cc (negativity central-contralateral) at C3/C4 electrodes (top panel) and N2pc (negativity posterior-contralateral) at PO7/PO8 electrodes (bottom panel) are shown for healthy elderly (thin solid line), sdaMCI (thin dashed line) and mdaMCI participants (thick dashed line)**. N2cc was calculated as [C4 − C3 (left hemifield stimuli) + C3 − C4 (right hemifield stimuli)]/2]. N2pc was calculated as [PO8 − PO7 (left hemifield stimuli) + PO7 − PO8 (right hemifield stimuli)]/2]. N2cc latency was delayed in mdaMCI relative to sdaMCI and healthy control group. N2pc amplitude was smaller in mdaMCI relative to healthy control group. Coefficient of N2cc/N2pc amplitudes did not differ between the three groups of participants.

The participants were instructed to direct their gaze towards the central cross throughout the task and they were instructed to respond to the color of the arrow, by pressing one of two horizontally positioned buttons (blue or red), but to ignore the position and the direction indicated by the arrow. The color of the arrows was appropriately discriminated by all the participants. The irrelevant dimensions (i.e., position and direction) gave rise to four experimental conditions depending on whether they were compatible or incompatible with the response to the color: compatible direction-compatible position (CDCP), incompatible direction-compatible position (IDCP), compatible direction-incompatible position (CDIP) and incompatible direction-incompatible position (IDIP). The same numbers of trials were run for all four conditions (80 per condition).

After a practice block of 24 trials (which was used to ensure that participants understood and were able of performing the task successfully), a total of 320 trials (80 per condition) were presented in two blocks, with an inter-block interval of 90 s The response button assigned to each color of the stimulus was counterbalanced among the participants, who were instructed to respond as quickly and accurately as possible.

### EEG Recordings

The EEG was recorded from 47 ring electrodes placed in an elastic cap (Easycap, GmgH), according to the International 10–10 system. The EEG signal was passed through a 0.01–100 Hz analog bandpass filter and sampled at 500 Hz. The reference electrode was placed on the tip of the nose and the ground electrode at Fpz. Simultaneously to EEG recordings, ocular movement (EOG) recordings were made with two electrodes located supra- and infraorbitally to the right eye (VEOG) and another two electrodes at the external canthus of each eye (HEOG). The impedance was maintained below 10 kΩs. As previous studies, a two-step procedure was used to remove epochs with horizontal ocular artifacts (e.g., Kiss et al., 2008). First, trials with large horizontal eye movements (larger than ±30 μV) were removed. Second, averaged HEOG waveforms showing residual eye movements (HEOG activity exceeding ±3 μV) were eliminated. All VEOG artifacts were also corrected off-line by use of the algorithm of Gratton et al. (1983). The signal was passed through a 0.01–30 Hz digital band-pass filter. Epochs with signals exceeding ±100 μV were automatically rejected, and all remaining epochs were inspected individually to remove those still displaying artifacts and exclude them from subsequent analyses.

The epochs were established from 200 ms pre-stimulus (baseline) to 1000 ms after stimulus presentation. The mean number of averaged epochs for each group was as follows: healthy elderly, 125; sdaMCI; 118; mdaMCI, 111, for the left hemifield, and healthy elderly, 122; sdaMCI, 122; mdaMCI, 108, for the right hemifield. Mixed measures ANOVA (Diagnosis × Hemifield) did not reveal any significant differences in number of epochs.

### Data Analyses

Trials with incorrect responses or RTs outside the 100–1500 ms range were excluded from the behavioral and ERP analysis.

The RT and the percentages of errors (PE) were analyzed by distinguishing between conditions (CDCP, IDCP, CDIP, IDIP) in order to obtain interference effects (i.e., interference from stimulus position and interference from arrow direction). However, distinction between conditions was omitted in ERP analyses.

N2pc and N2cc were obtained on the basis of the hemifield of stimulus presentation (e.g., Woodman and Luck, 1999): N2pc = [PO8 − PO7 (left hemifield stimuli) + PO7 − PO8 (right hemifield stimuli)] / 2]; N2cc = [C4 − C3 (left hemifield stimuli) + C3 − C4 (right hemifield stimuli)] / 2] irrespective of whether stimulus position and arrow direction were compatible or incompatible with the required response. This method does not allow comparison between conditions. However, it has the advantage that residual motor activity is removed from N2cc and N2pc waveforms. Specifically, half of the arrows located in the left hemifield require a left-handed response, whereas the other half requires a right-handed response. Averaging across all these trials, motor activity is cancelled out. However, as the target is always located in the left hemifield, target-related activity (i.e., N2cc and N2pc) remains in these waveforms. The same reasoning can be applied to averages for right-hemifield stimuli.

The N2pc and N2cc peak latencies were identified as the largest negative peaks between 200–400 ms after stimulus presentation. The average amplitude of N2pc and N2cc was calculated, for each participant, as ±20 ms around peak latency.

### Statistical Analyses

RT and PE were analyzed by using the corresponding mixed measures ANOVA with two within-subject factors: Position (two levels: Compatible, Incompatible) and Direction (two levels: Compatible and Incompatible) and one between-subject factor: Diagnosis (three levels: Control Group, sdaMCI and mdaMCI).

The corresponding univariate ANOVA with one between-subject factor, Diagnosis (three levels: CG, sdaMCI and mdaMCI), was carried out to study MCI-related changes in latencies and amplitudes of the N2cc and negativity posterior contralateral (N2pc) as well as in direct visuomotor transmission (obtained by calculating N2cc/N2pc coefficient on each individual participant). Those ERP parameters showing differences according to the Diagnosis factor were examined by Receiver Operating Characteristic (ROC) curve analysis, which yields area under curve (AUC), cut-off, sensitivity and specificity values.

Pearson’s correlation analysis was carried out to study correlations between ERP parameters (N2cc latency and amplitude and coefficient of direct visuomotor transmission, i.e., N2cc/N2pc) and behavioral data (RT and PE and stimulus position interference, which was calculated by subtracting spatially incompatible trials from spatially compatible trials). Pearson’s correlation analysis was also carried out to study correlations between the behavioral/ERP parameters and age.

A Greenhouse-Geisser ε correction for the degrees of freedom was performed in all cases where the condition of sphericity was not met, and in these cases the corresponding α levels are provided. Measures of size effect (eta square - $\eta_{\rho }^{2}$ -) are also provided for significant results. When the ANOVAs revealed significant effects due to the factors and their interactions, *post hoc* multiple paired comparisons (with Bonferroni adjustment) of the mean values were carried out.

## Results

### Behavioral Measures

Regarding behavioral changes related to MCI (see Table 2), the mixed measures ANOVA (Position × Direction × Diagnosis) revealed a Position effect (*F*_(1,40)_ = 119.6, *p* < 0.001, $\eta_{\rho }^{2}$ = 0.749), as the RT was longer when the stimulus position was incompatible than compatible with the required response (*p* < 0.001).

**Table 2:** **Recap of behavioral and event-related potential (ERP) results (i.e., mean values ± standard error of the mean, in parenthesis) obtained in healthy elderly (Control Group, CG), single-domain amnestic Mild Cognitive Impairment (sdaMCI) and multiple-domain amnestic Mild Cognitive Impairment (mdaMCI)**.

	CG	sdaMCI	mdaMCI
**Total RT**	**594.6 (24.3)**	**614.1 (28.6)**	**635.4 (29.7)**
RT_CDCP	567.5 (23.1)	583.3 (27.2)	611.2 (28.2)
RT_IDCP	568.5 (24.1)	596.9 (28.3)	607.4 (29.5)
RT_CDIP	624.8 (25.8)	636.2 (30.4)	667.3 (31.6)
RT_IDIP	617.8 (26.1)	639.8 (30.7)	655.7 (31.9)
**Total PE**	**4.9 (0.92)**	**3.7 (1.09)**	**9.0 (1.14)**
PE_CDCP	2.6 (0.86)	1.8 (1.00)	5.1 (1.01)
PE_IDCP	2.2 (0.82)	2.1 (0.97)	5.9 (1.00)
PE_CDIP	8.1 (1.37)	5.4 (1.61)	12.2 (1.68)
PE_IDIP	6.7 (1.32)	5.7 (1.56)	12.6 (1.62)
**N2cc lat**	**286.8 (7.16)**	**260.4 (8.43)**	**336.2 (8.78)**
**N2cc amp**	**−2.6 (0.32)**	**−1.7 (0.38)**	**−2.0 (0.40)**
**N2pc lat**	**294.7 (7.46)**	**284.7 (8.78)**	**312.0 (9.14)**
**N2pc amp**	**−2.9 (0.35)**	**−2.0 (0.41)**	**−1.0 (0.43)**

*Values provided without distinguishing among conditions are highlighted in bold. Behavioral data (reaction times [RT] and percentage of errors [PE]) are provided differencing between conditions (Compatible Direction-Compatible Position –CDCP- Incompatible Direction-Compatible Position –IDCP- Compatible Direction-Incompatible Position –CDIP- and Incompatible Direction-Incompatible Position -IDIP) as well as averaging across conditions. Latencies (in milliseconds) and amplitudes (in μV) for negativity central-contralateral (N2cc) and negativity posterior-contralateral (N2pc) are also reported*.

The mixed measures ANOVA (Position × Direction × Diagnosis) for the PE revealed a Diagnosis effect (*F*_(2,40)_ = 6.2, *p* = 0.005, $\eta_{\rho }^{2}$ = 0.234), as PE was higher in mdaMCI participants than in CG (*p* = 0.026) and sdaMCI participants (*p* = 0.006). An effect of the Position was also revealed (*F*_(1,40)_ = 62.1, *p* < 0.001, $\eta_{\rho }^{2}$ = 0.608), as PE was higher when stimulus position was incompatible with the response.

### ERP Measures

For N2cc latency (see top panel of Figure 1; Table 2), the univariate ANOVA (Diagnosis) revealed a significant effect (*F*_(2,40)_ = 19.9, *p* < 0.001, $\eta_{\rho }^{2}$ = 0.499), as the N2cc latency was longer in mdaMCI than in CG (*p* < 0.001) and sdaMCI participants (*p* < 0.001). N2cc amplitude did not reveal any differences according to the Diagnosis factors. Univariate ANOVA (Direct visuomotor transmission) did not reveal a significant effect (*F*_(2,40)_ = 1.24, *p* = 0.30, $\eta_{\rho }^{2}$ = 0.058).

For N2pc latency, the univariate ANOVA (Diagnosis) did not reveal any significant effects. For N2pc amplitude (see bottom panel of Figure 1; Table 2), the univariate ANOVA (Diagnosis) revealed a significant effect (*F*_(2,40)_ = 6.14, *p* = 0.005, $\eta_{\rho }^{2}$ = 0.235), as the N2pc amplitude was smaller in mdaMCI compared to healthy participants (*p* = 0.005).

**Figure 2:**
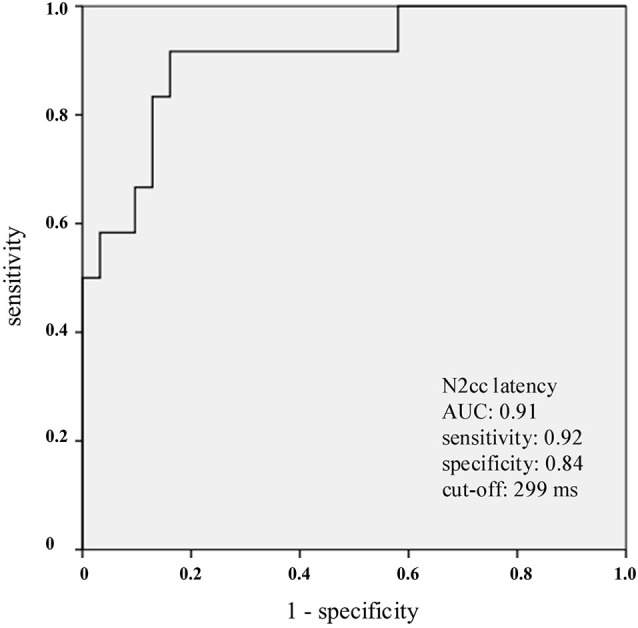
**Receiver operating characteristics (ROC) curves for N2cc latency**. N2cc latency clearly distinguished the mdaMCI participants (positive group) from the control group (CG) and sdaMCI participants (negative groups) As indicated in the graph, and considering 299 ms as the cut-off, the sensitivity and specificity values were 0.92 and 0.84, respectively.

ROC analysis of N2cc latency (which compared mdaMCI vs. sdaMCI and CG) yielded an AUC of 0.91 (see Figure 2). Considering 299 ms as cut-off, the indexes of sensitivity and specificity were 0.92 and 0.84, respectively. Also, ROC analysis of N2pc amplitude (which compared mdaMCI vs. CG) yielded an AUC of 0.78. Considering −2.25 μV as cut-off, the indexes of sensitivity and specificity were 0.91 and 0.62, respectively.

Pearson correlation analysis revealed a correlation between N2cc latency and PE (*r* = 0.416, *p* = 0.006). N2pc and N2cc amplitudes were not significantly correlated with behavioral parameters; however, the direct visuomotor transmission index (N2cc/N2pc coefficient) was correlated with PE (*r* = 0.376, *p* = 0.013). Although the age range was narrow (60–83), some significant correlations between behavioral/ERP parameters and age were obtained. Specifically, there was a significant correlation between age and the N2cc/N2pc coefficient (*r* = 0.303, *p* = 0.049). Age and PE were also correlated (*r* = 0.422, *p* = 0.005).

## Discussion

In the present study, samples of healthy elderly, single-domain amnestic MCI (sdaMCI) and multiple-domain amnestic MCI (mdaMCI) participants performed a Simon task while EEG was recorded. The main aim of the study was to identify a correlate of cognitive control (the negativity central-contralateral, N2cc) that could be associated with the increased interference usually observed in individuals with MCI. The results showed greater interference in mdaMCI than in sdaMCI and healthy elderly participants, as revealed by a higher PE in mdaMCI than in the other groups of participants. The N2cc latency was also longer in mdaMCI than in healthy elderly and sdaMCI participants. The AUC was 0.91, providing sensitivity and specificity values of 0.92 and 0.84, respectively.

Behavioral performance revealed a deficit in monitoring the selection of the correct response in mdaMCI participants while performing the Simon task. Specifically, the PE was higher in mdaMCI than in sdaMCI and healthy elderly participants. This result was consistent with previous reports of the effects of MCI in Simon-type tasks (Castel et al., 2007; Cespón et al., 2013a; Pereiro et al., 2014) and in studies using other cognitive tasks (Wylie et al., 2007; Lindín et al., 2013; Cid-Fernández et al., 2014). These findings are also consistent with reported deficits in executive processes in mdaMCI (Johns et al., 2012), which increase in AD (Perry and Hodges, 1999; Amieva et al., 2004). On the other hand, the lack of interference in sdaMCI participants is consistent with preserved executive functioning in this group of participants.

Regarding the experimental manipulation, the behavioral results revealed the typical Simon effect, i.e., longer RT and higher PE when stimulus position was spatially incompatible with the required response compared to trials in which stimulus position was spatially compatible with the required response. Such findings have been widely described in studies using Simon tasks in healthy young (Lu and Proctor, 1995; Leuthold, 2011), healthy elderly (Proctor et al., 2005; Juncos-Rabadán et al., 2008), and MCI participants (Cespón et al., 2013a; Pereiro et al., 2014).

The ERP data provided evidence for the mechanisms underlying the increased interference in mdaMCI participants. In particular, the N2cc component, a correlate of processes implemented to prevent cross-talk between the direction of the spatial attention and the manual response preparation (Oostenveld et al., 2001; Praamstra and Oostenveld, 2003; Praamstra, 2006), was delayed in mdaMCI relative to sdaMCI and healthy elderly participants. The longer N2cc latency observed in mdaMCI participants suggests delayed activity related with attention in dPM in order to prevent the preparation of the response on the basis of the stimulus position (as it was incorrect in a half of the trials). Further evidence for a link between delayed N2cc latency and increased PE in mdaMCI participants was provided by the significant correlations between N2cc latency and PE. On the other hand, a correlation between RT and N2cc latency was not found. This result suggests that neural mechanisms related with N2cc (i.e., mechanisms to prevent spatial tendencies of response on the basis of the stimulus position) emerge in parallel to stimulus processing. Also, considering that N2cc is a stimulus-locked component, this result is consistent with studies showing a relationship between slowing in RT and slowing in motor execution processes during Simon-type tasks (Kolev et al., 2006; Cespón et al., 2013b).

The N2cc latency clearly distinguished mdaMCI individuals from sdaMCI and healthy participants. Considering 299 ms as the cut-off, N2cc latency provided sensitivity and specificity indexes of 0.92 and 0.84, respectively. For consideration of N2cc latency as a possible early biomarker of AD, follow-up studies should consider the predictive usefulness of N2cc latency to differentiate between participants who progress and participants who do not progress to AD. On the other hand, the absence of a delay in N2cc latency observed in sdaMCI participants was consistent with the absence of increased interference effect in this group. Thus, sdaMCI did not show behavioral deficits in cognitive control and neither in electrophysiological correlates of cognitive control. This result suggests that brain mechanisms underlying cognitive control in this type of tasks are preserved in sdaMCI participants. It was pointed that deficits in brain mechanisms precede behavioral manifestations (Galli et al., 2010; Vallesi and Stuss, 2010); nonetheless, on the basis of the present evidence, the possibility of sdaMCI participants exhibiting incipient covert deficits in cognitive control of automatic response spatial tendencies may be discarded.

In the present study, the difference in N2cc latency provided evidence for mechanisms underlying executive function deficits in mdaMCI participants. In this context, previous studies had related N2cc to the “ancillary monitoring mechanism” (Leuthold and Schröter, 2006; Cespón et al., 2012), a cognitive control mechanism involved in selecting the correct response and suppressing the non-correct response in Simon-type tasks (Stürmer and Leuthold, 2003). Overall, inhibition of preeminent responses in SRC tasks was considered to reflect executive control mechanisms, which promotes correct and flexible responses (Miller and Cohen, 2001; Ridderinkhof et al., 2004). Thus, the present findings establish a link between delays in implementing executive processes and increased PE in mdaMCI participants who were performing a Simon task.

In consistence with a previous study (Cespón et al., 2013a), the amplitude of N2pc was smaller in mdaMCI compared to CG. These results suggest a reduced allocation of attentional resources to the target stimulus in mdaMCI participants, which is also consistent with behavioral evidence for declined visuospatial abilities in MCI (Iachini et al., 2009). Also, N2pc amplitude fairly distinguished mdaMCI from healthy elderly participants. In fact, ROC analysis showed an AUC of 0.78. Thus, N2pc amplitude represents a good index to distinguish mdaMCI from healthy elderly.

Visuomotor transmission from parietal to central areas (which is revealed by N2cc/N2pc coefficient) was correlated with increased PE. This result point to disinhibition of a parieto-precentral pathway as a mechanism underlying to increased PE (in addition to the relationship between N2cc latency and PE observed in mdaMCI participants). Furthermore, although the age range of the participants was fairly narrow (60–83 years old), increased PE and direct visuomotor transmission were positively correlated with age, which newly provided evidence for linking age-related increase in PE to parieto-precentral desinhibitory processes. In addition, these findings are consistent with those that related increased PE with aging and increased direct visuomotor transmission (van der Lubbe and Verleger, 2002; Amenedo et al., 2012), and they are also similar to the results of a study focusing on changes related to Parkinson’s disease (Praamstra and Plat, 2001). However, in the present study, increased PE in mdaMCI was related to delays in implementing executive control processes (as N2cc was longer in mdaMCI than in sdaMCI and CG) but not to increased direct visuomotor transmission (as N2cc/N2pc coefficients were not different in healthy control elderly, sdaMCI and mdaMCI participants). Thus, increased PE in healthy aging and Parkinson’s disease may be explained by different underlying mechanisms whereby PE increased in mdaMCI participants.

In summary, in the present study samples of healthy elderly, sdaMCI and mdaMCI participants performed a Simon task while EEG activity was recorded. Behavioral performance evidenced increased interference in mdaMCI relative to healthy elderly and sdaMCI participants, as revealed by higher PE in the mdaMCI participants. The increased interference was associated with delays in implementing cognitive control in mdaMCI participants, which was indicated by longer N2cc latencies in this group. On the basis of N2cc latency, mdaMCI participants were clearly distinguished from healthy elderly and sdaMCI participants (AUC: 0.91). Considering 299 ms as a cut-off, N2cc latency provided sensitivity and specificity values of 0.92 and 0.84 respectively. Therefore, the present study provides evidence for considering N2cc as a useful mdaMCI correlate. Follow-up studies should investigate the predictive utility of N2cc latency to discriminate between participants who will develop AD and participants whose condition will remain stable.

## Authors Contributions

JC: Contributed to the design of the work, acquisition, analysis and interpretation of the results for the work as well as drafting the work. SG-A: Contributed to the design of the work, analysis and interpretation of the results for the work as well as revising critically the work. FD: Contributed to the design of the work and interpretation of the results for the work as well as revising critically the work. All the authors agree to be accountable for all aspects of the work in ensuring that questions related to the accuracy or integrity of any part of the work are appropriately investigated and resolved. All the authors approved the version to be published.

## Conflict of Interest Statement

The authors declare that the research was conducted in the absence of any commercial or financial relationships that could be construed as a potential conflict of interest.
